# A replication of the study ‘Adverse effects of spinal manipulation: a systematic review’

**DOI:** 10.1186/2045-709X-20-30

**Published:** 2012-09-21

**Authors:** Peter Tuchin

**Affiliations:** 1 Macquarie University, Bld E5A Rm 355, Waterloo Rd, North Ryde, Sydney, NSW, 2109, Australia

**Keywords:** MeSH, Chiropractic, Spinal manipulative therapy, Adverse effects

## Abstract

**Objective:**

To assess the significance of adverse events after spinal manipulation therapy (SMT) by replicating and critically reviewing a paper commonly cited when reviewing adverse events of SMT as reported by Ernst (J Roy Soc Med 100:330–338, 2007).

**Method:**

Replication of a 2007 Ernst paper to compare the details recorded in this paper to the original source material. Specific items that were assessed included the time lapse between treatment and the adverse event, and the recording of other significant risk factors such as diabetes, hyperhomocysteinemia, use of oral contraceptive pill, any history of hypertension, atherosclerosis and migraine.

**Results:**

The review of the 32 papers discussed by Ernst found numerous errors or inconsistencies from the original case reports and case series. These errors included alteration of the age or sex of the patient, and omission or misrepresentation of the long term response of the patient to the adverse event. Other errors included incorrectly assigning spinal manipulation therapy (SMT) as chiropractic treatment when it had been reported in the original paper as delivered by a non-chiropractic provider (e.g. Physician).

The original case reports often omitted to record the time lapse between treatment and the adverse event, and other significant clinical or risk factors. The country of origin of the original paper was also overlooked, which is significant as chiropractic is not legislated in many countries. In 21 of the cases reported by Ernst to be chiropractic treatment, 11 were from countries where chiropractic is not legislated.

**Conclusion:**

The number of errors or omissions in the 2007 Ernst paper, reduce the validity of the study and the reported conclusions. The omissions of potential risk factors and the timeline between the adverse event and SMT could be significant confounding factors. Greater care is also needed to distinguish between chiropractors and other health practitioners when reviewing the application of SMT and related adverse effects.

## Introduction

The use of a treatment by health care providers requires examination of the evidence of effectiveness and assessment of the evidence for risks or adverse events (AE) caused by the treatment [1]. Spinal manipulation therapy (SMT) has strong evidence for treatment of low back pain, neck pain, headache and migraine [2-6]. This is supported by numerous systematic reviews of a large number of randomized controlled trials [7-10].

Adverse events following SMT are common but usually result in minor, short term problems [11-14]. The most commonly expressed area of concern regarding these adverse effects of spinal manipulation includes conditions such as vertebral artery dissection (VAD) or carotid artery dissection (CAD), which results in cerebrovascular accidents (CVA) [15]. These serious AE can occur immediately following SMT but are reported as rare and unpredictable consequence of all neck movements [16]. However, there appears to be conflict over the frequency or severity of serious events which may follow SMT. A paper written by Ernst “Adverse effects of spinal manipulation: a systematic review” was published in the Journal of the Royal Society of Medicine in July 2007 [17]. In the paper, 28 articles reporting a total of 32 case reports were identified with 22 cases involving chiropractors. The majority of cases involved complications from cervical manipulation, the most notable problem being VAD. According to the 2007 Ernst paper, “the symptoms were frequently life-threatening”, and in the majority of cases “spinal manipulation was deemed to be the probable cause of the adverse effect” [17]. In addition, SMT applied by chiropractors was specified by a separation of the papers noting chiropractic as the cause of the AE.

It is important that all the clinical features for these AE are clearly identified and recorded, with any potential predisposing risk factors described [14]. As the 2007 Ernst paper is commonly cited when assessing any risk with cervical spine SMT, it may be regarded as a significant paper [18]. This paper is a replication of the 2007 Ernst paper, and a critical review of the initial results reported in the 2007 paper.

## Methods

The case reports in the reference list from the 2007 Ernst paper were retrieved and reviewed in detail. The clinical details of each case were then cross-checked with Ernst’s paper for accuracy and relevance towards assessing adverse events. Particular interest was paid towards papers that involved chiropractic SMT and its subsequent effects.

Each paper was reviewed to assess significant risk factors for CVA that had been identified in the literature [18,19]. These include factors such as use of oral contraceptive pill (OCP), any history of hypertension, atherosclerosis, family history of cardiac conditions, smoking, previous headaches or migraine, and genetic factors [19-24]. Also, each paper was reviewed to assess the likelihood that the provider that rendered the treatment was a qualified chiropractor. This was determined by a review of the details provided describing the SMT procedure, and by reviewing the country of origin for the case report. Any description of the SMT was assessed to determine if this was a commonly performed chiropractic procedure or if it described SMT commonly performed by other practitioners.

In addition, other items that were assessed included the description of the presenting symptom(s), the time lapse between treatment and the type of adverse event. This included examining any diagnosis of the presenting symptom to the SMT practitioner, the onset of symptoms relating to the adverse event, and assessing any other events that could be a confounding factor for assessment of the relationship between SMT and the adverse event.

Papers from the 2007 Ernst study were excluded if the abstract or full text could not be obtained.

## Results

In the 2007 Ernst paper, 28 articles reporting a total of 32 case reports were identified with 22 cases involving chiropractors. Case reports where the paper was not published in English often had only an abstract which could be reviewed. The outcome of these papers is presented as separate issues.

The review of the original case reports and case series papers described by Ernst found numerous errors or inconsistencies (See Table 1- Errors Or Inconsistencies In The 2007 Ernst Paper). These errors included alteration of the age or sex of the patient, and omission or misrepresentation of the long term response of the patient to the adverse event.

**Table 1:** Errors or Omissions to 2007 Ernst paper

**Author**	**Presenting symptoms**	**Outcome**	**Comments**	**Error or omission**
**Chiropractic**				
Jeret	Not noted	Complete resolution	Dural tear vs IH inconsistency	
Siegel	HA- several weeks, no IHS categories noted;	Permanent	Letter to the editor	
Parwar	Shoulder strain	Horner’s syndrome; no further neurologic abnormalities developed	9 previous, uneventful treatments	noted as male; outcome “not reported“
Schram	Neck pain and stiffness after sleeping on sofa	Shortness of breath on exertion and breathing in the supine position	NB ref 12 notes stretch trauma;	Residual dyspnea
Stevinson	Unknown	Complete resolution after surgery	Subdural haematoma; Recall cases over 12mths	
Jeret	Neck pain, OCP	Complete resolution		
	Unknown	Flow re-established after recanulization		Outcome “not mentioned”
	Unknown	Horner’s syndrome (opposite side) after 2 weeks hospital	new Horner’s syndrome	“significant recovery after surgery”
Jay	4 day HA, fever, cough, sinusitis, antibiotics	Complete resolution	Polycysytic ovarian disease	Outcome “not mentioned”
Nadgir	Neck cramping	Minimal residual hemianasthesia and dysethesiae	B/L ICA & VAD	“residual hemianasthesia and dysethesiae”
**Non chiropractic**				
Se’dat	Chronic headaches, anti-inflam and pain medication	Positive outcome after 3 weeks	Genetic variation	“mild” removed from outcome statement
Beck	Unknown	Complete resolution confirmed by MRI	IH but reported as Wallenberg’s syndrome,	“no information provided”; not Wallenberg’s syndrome
Oehler	Unknown, past HA	Resolution	10yrs chiro and HA	Outcome “not mentioned”
Morandi	CLBP, aggravated by heavy lifting	Permanent neuro loss	CLBP with acute episode	Noted as 44 yo,
Saxler		Complete resolution	no translation	
Chen	Neck pain- relieved by chiropractor		Haematoma after massage	Massage therapy
Suh	Chronic neck and shoulder pain)	Complete resolution	IH	Reported as 36 yo female
**Abstract only**				
Yokota	Unknown	Dejerine syndrome	no translation	
Izquierdo-Casas	Unknown		no translation	
Menendez-Gonzalez	Unknown	Wallenberg’s syndrome,	no translation	
Tome	Unknown	Multiple cervical disc lesions	no translation	
Wojcik		Publication withdrawn		

More serious concerns involved the interpretation of clinical features for each case and the subsequent conclusions regarding the safety of chiropractic SMT. For example, Table 1 in the 2007 Ernst paper summarizes case reports of AE after SMT administered by chiropractors. However, two case reports clearly state the SMT was administered by non-chiropractors. Morandi reported a 49 year old female that developed caudal spinal cord ischaemia after a lumbar SMT [25]. The paper state’s “*Three weeks into the episode, a physician performed lumbar vertebral manipulation*”. Also, Chen reported a 72 year old male that developed a haematoma in the ligamentum flavum [26]. The paper states “….*following traditional massage therapy*”.

Nine cases originated from countries where there is no legislation governing chiropractic, which therefore allows non-qualified people to administer SMT, and this may be incorrectly termed “chiropractic” [27-35]. In four of these cases only the abstract was available in English, so a less detailed review could be performed for these papers. However, in the seven cases with full English papers, a detailed review is discussed later in this paper.

Therefore, in 11 cases of the 21 (one paper has been withdrawn from circulation) that Ernst reported as SMT administered by chiropractors, it is unlikely that the person was a qualified chiropractor. Whilst most cases did not describe the SMT procedure, the three cases which did, describe SMT procedures not commonly used by chiropractors [25,28,30]. For example, facet joint infiltration after the SMT and SMT with axial traction, which was initially described by Cyriax (a medical practitioner) [36]. This supports the theory that these 11 cases had SMT from a non-chiropractor, which has previously been reported [37]. (See Table 2: Countries With Legislation For Chiropractic) .

**Table 2:** Reference papers and their country of origin and relevant legislation for chiropractic licensing

**Reference**	**Country**	**Legislation regulating chiropractic**
[25]	France	No
[26]	China/Taiwan	No
[27]	France	No
[28]	German (English translation)	No
[29]	German (in german)	No
[30]	German	No
[31]	Korea	No
[32]	Japan (in japanese)	No
[33]	Spain (in spanish)	No
[34]	Spain (in spanish)	No
[35]	Spain (in spanish)	No
[38]	USA	Yes
[39]	USA	Yes
[40]	USA	Yes
[41]	USA	Yes
[42]	Unknown –survey of UK neurologists	Yes
[43]	USA	Yes
[44]	USA	Yes
[45]	USA	Yes
[46]	USA	Yes
	USA	Yes

Importantly, in five of the seven cases (with full English versions available) complete resolution of the AE was reported after appropriate treatment. Also, three of the seven cases describe the time interval following SMT to the onset of symptoms as greater than 24 hours (i.e. 24 – 96 hours). Given that many VAD occur spontaneously or due to simple everyday activities, a time interval of greater than 24 hours is not appropriate.

In the remaining 10 cases it is more likely that the person was a qualified chiropractor [38-44]. However, as most of these cases (n = six) did not describe the SMT procedure, it cannot be determined if the SMT procedure was a type commonly used by chiropractors. In addition, there is the possibility of poor execution of the SMT procedure or inappropriate use of the procedure (i.e. professional negligence).

Many of these 10 cases also had a plausible alternative explanation as to why the AE may have occurred (See Table 3: Features And Outcomes Of Cases). For example, Jeret described a case of a 51-year-old man who presented to the hospital five days after rotatory chiropractic manipulation of his neck [38]. For 2–3 days prior to admission, the patient was reported to have had intermittent slurred speech, left facial droop, and mild left hand weakness. The case had no description of the presenting symptom and no description of any risk factors for CVA. Interestingly, the initial symptoms were all related to a left side infarct and 2 weeks after admission to hospital the patient had a right side infarct. It is plausible this man had CVA due to risk factors other than chiropractic SMT. 

**Table 3:** Features And Outcomes Of Cases

**Author**	**Patient**	**Presenting symptoms**	**SMT Procedure**	**Time after SMT for onset of symptoms**	**Additional information**
Jeret	34 yo male	Not noted	Not noted	36 hrs after	Recent MVA
Siegel	33 yo female	HA- several weeks, no IHS categories noted;	HVLA	immediate	
Parwar	44yo female	Shoulder strain	Not noted	Several days	9 previous CSMT without incident
Schram	47yo male	Neck pain and stiffness after sleeping on sofa	Shoulders forced down and turning head laterally	Later at night; initial relief by SMT	Neck position whilst sleeping
Stevinson	46yo male	Unknown	Unknown	immediate	Subdural haematoma from previous unreported trauma
Jeret	31yo female	Neck pain,	Rapid rotation	immediate	Complication from OCP
	64 yo male	Unknown	Gentle SMT (daughter DC)	4 days	Age related CVA
	51 yo male	Unknown	rotation	2-3 days	
Jay	26 yo female	4 day HA, fever, cough, sinusitis, antibiotics	20 previous DC treatments-not described	36hrs	Complication from inflammatory response
Nadgir	34 yo male	Neck cramping	Several previous sessions, not described	Immediate, but symptoms not described	Neck cramping a symptom of impending CVA
**Non chiropractic**					
Se’dat	42 yo female	Chronic headaches, anti-inflammatory and pain medications	Gentle SMT	immediate	Complication from inflammatory response, Un-registered practitioner
Beck	40 yo female	Unknown	Axial tension and rotation- (Cyriax manoeuver)	24 hrs	Not chiropractic SMT
Oehler	31 yo female	Unknown, past HA	Unknown, previous regular chiropractic	Minimum of 24 hrs	Un-registered practitioner
Morandi	49 yo female	CLBP, aggravated by heavy lifting	Physician	Few hours	Not chiropractic SMT
Saxler	27 yo female		C5/6 SMT, facet joint infiltration	10 min	Not chiropractic SMT
Chen	72 yo male	Neck pain- relieved by chiropractor	Massage therapy	1 week, symptoms began after hospital treatment	Not chiropractic SMT
Suh	37 yo female	Chronic neck and shoulder pain)	Cyriax treatment (reported as “chiropractic manoeuver”)	5 days	Not chiropractic SMT
**Abstract only**			**probably not chiropractic**		
Yokota	38 yo male	Unknown	Unknown	Unknown	Un-registered practitioner
Izquierdo-Casas	37 yo female	Unknown	Unknown	Unknown	Un-registered practitioner
Menendez-Gonzalez	Not noted	Unknown	Not noted	A few hours	Un-registered practitioner
Tome	Not noted	Unknown	“non qualified personnel”	Unknown	Un-registered practitioner

Key: *IH* = intracranial hypotension; *HVLA* = high velocity, low amplitude; *OCP* = oral contraceptive pill; *HA* = headache; *IHS* = international headache society; *B/L* = bilateral; *CVA* = internal carotid artery; *VAD* = vertebral artery dissection; *CLBP* = chronic low back pain; *SMT* = spinal manipulative therapy.

Parwar (2001) reported a case of a 44-year-old woman who presented to an emergency room with severe headache, right-sided neck pain, ptosis, and miosis [40]. It was reported that this happened several days after her ninth chiropractic session for her presenting symptom of “a strained right shoulder muscle”. The patient was diagnosed with Horner’s Syndrome due to a right internal carotid artery dissection. None of the risk factors for CVA were reported, for example, migraine, which has been reported to cause Horner’s Syndrome [19]. The case report also stated that no further neurological abnormalities occurred, which Ernst reported as “not reported”.

Schram reported a case of a 41-year-old male who sought chiropractic care for pain and stiffness in his neck and shoulders [41]. The pain developed after the patient had slept on a sofa while on vacation, one week previous to the treatment. The case report stated the “chiropractor did a number of manipulations that the patient described as forcing his shoulders downward and turning his head laterally” (not a common chiropractic technique). There was no record that the patient experienced increased neck pain or any alteration in breathing pattern. He returned to his chiropractor the next day with this complaint, and was subsequently transferred to an emergency department for evaluation of his orthopnea.

Stevinson (2001) described a case of a 46 year old male that was reported to suffer a subdural haematoma after a chiropractic treatment [42]. The case was obtained from a survey to UK neurologists, who were asked to recall patients that had a neurological complication within a 12 month period. The entire case history is reproduced below: 

"A man aged 46 was diagnosed with acute subdural haematoma occurring immediately after chiropractic treatment. A burrhole was required. There was no neurological deficit at one month or six month follow up."

Apart from the obvious weakness of recall bias with the survey, the case details could at best only be described as scant. It is more likely this is a case of negligence, with the chiropractor failing to adequately investigate any previous trauma that would have caused the subdural haematoma. This would then become a contraindication for SMT and require immediate referral for emergency medical treatment.

Jeret reported a case of a 34 year old male who had a whiplash injury in a motor vehicle accident (MVA), for which chiropractic treatment gave some relief [43]. One month after the MVA, the chiropractor performed a SMT and approximately 36 hours after this the patient reported a severe, throbbing, positional headache. The clinical diagnosis was intracranial hypotension, which is commonly reported as spontaneous.

Jay et al (2003) described a case of bilateral occipital-parietal hemorrhagic infarctions following chiropractic cervical manipulation [44]. A 26-year-old woman presented to a chiropractor with mild headache, cough, and low-grade fever for four days (for which she had been prescribed antibiotics). The patient had received over 20 chiropractic manipulations over the previous two years without event. CT scan and MRI were reported to show bilateral, symmetric occipital-parietal hemorrhagic infarctions. The patient was taking the OCP (for polycystic ovarian disease), however, other risk factors for stroke were not recorded. In addition, the time between onset of visual changes and SMT was over 36 hours. It is plausible this woman had CVA due to risk factors other than chiropractic SMT. The patient also made a full recovery.

## Discussion

The general quality of the original case information used by Ernst was poor, which should have called into question the conclusions published in the 2007 Ernst paper. The lack of clinical detail in the original cases does not allow adequate conclusions to be drawn from these cases. For example, the vast majority of the original cases did not contain information about well-established confounding variables for these types of cases. Subsequently, no conclusions can be correctly derived about the impact of SMT on the development of VAD. The author of the 2007 paper should have concluded previous cases material was inadequate for any relationship between SMT and VAD to be assessed.

The terms chiropractic treatment, chiropractic manipulation, chirotherapy and/or chiropractic procedure were used repeatedly, without identification of the practitioners’ qualifications. A study by Terrett found that “the words chiropractic and chiropractor have been incorrectly used in numerous publications dealing with SMT injury by medical authors, respected medical journals and medical organisations. In many cases, this is not accidental; the authors had access to original reports that identified the practitioner involved as a non-chiropractor” [36]. Wenban also suggests that the words ‘chiropractor’ and ‘chiropractic manipulation’ are used inappropriately by European biomedical researchers [45]. This appears to be continuing with the Ernst’s paper giving the impression that most AE after SMT are due to chiropractic procedures “….(SMT) is the hallmark treatment of chiropractors”.

Another weakness in many of the original case studies involved poor information on the time period between the onset of the AE and the administration of the SMT. Few cases described any events which may have occurred after the SMT that could have had a direct bearing on the subsequent AE occurrence. For example, one person drove home after her treatment and whilst she was reversing the car, the AE occurred. The sustained neck rotation could have had more impact than the SMT.

There were also further examples were plausible alternative processes may have led to the development of a VAD, which was independent to the SMT. For example, Ernst failed to acknowledge the anatomical variance in the patient’s vertebral arteries causing them to originate below the level of the foramen magnum which the author acknowledges to have played a vital role in the patient’s complications following spinal manipulative therapy [27].

There was also no discussion on the relevance of time lapse between SMT and the AE, where, in some cases, periods of greater than 24 hours occurred. This combined with many cases not describing the presenting symptom, creates significant weaknesses in establishing a link of the AE to SMT.

## Conclusion

It is unwise to make conclusions regarding causality from any case study or multiple case studies. The number of errors or omissions in the 2007 Ernst paper significantly limit any reported conclusions. Standard risk factors for VAD and the timeline between the VAD and SMT could be significant confounding factors. Whilst SMT may coincide with development of VAD, the quality of the 2007 paper does not add to the understanding of whether there is any link of SMT to VAD. A much more thorough peer review needs to occur regarding papers such as Ernst 2007. Further research is required to assess the relationship between SMT and VAD.

## Competing interests

The author declare that they have no competing interests.

## Authors’ information

Peter Tuchin: Senior Lecturer.
